# The Role of Spinal Instability in Treating Unstable Infected Fractures

**DOI:** 10.7759/cureus.78966

**Published:** 2025-02-13

**Authors:** James Garda, Prashant Shah, Awais Z Vance

**Affiliations:** 1 Neurosurgery, Baylor College of Medicine, Temple, USA; 2 Cardiothoracic Surgery, Baylor Scott and White Medical Center - Temple, Temple, USA; 3 Neurosurgery, Baylor Scott and White Medical Center - Temple, Temple, USA

**Keywords:** cobalt chromium, fracture-related infection, spinal fracture fixation, spinal instability, spinal stability, stainless steel, titanium implant, vertebral discitis, vertebral osteomyelitis

## Abstract

Vertebral (spinal) osteomyelitis is a rare spinal infection of the vertebral column that can be caused by bacteria or fungi. Though the initial treatment for all patients with vertebral osteomyelitis consists of antibiotics, surgery can be indicated in certain cases, such as in the presence of spinal instability or when antibiotics are not successfully eliminating the infection. Achieving spinal stability can be an important component of treating fracture-related infections, as instability can cause local damage and disrupt the healing process. The authors present the case of a patient with vertebral osteomyelitis that was not resolving with the administration of antibiotics in the context of a spinal fracture. A minimally invasive surgery was performed to achieve spinal stability and the infection soon resolved with antibiotics.

## Introduction

Vertebral osteomyelitis is a rare spinal infection of the vertebral column that is caused by bacteria or fungi. It typically develops from hematogenous dissemination of organisms from nearby tissue, though it can also occur due to spinal injury, spinal surgeries, or epidural injections [1]. *Staphylococcus aureus* is the most common pathogen causing vertebral osteomyelitis overall, followed by *Escherichia coli *[2]. *S. aureus* is the most common pathogen in cases of hematogenous spread, whereas coagulase-negative staphylococci and *Propionibacterium acnes* are the most common pathogens in cases of exogenous osteomyelitis that develops after spinal surgery, especially with the use of spinal fixation devices [1]. Vertebral osteomyelitis comprises 3%-5% of all osteomyelitis cases annually, with incidence in the United States standing at 4.8 cases per 100,000 people. One-year mortality can be as high as 11% [1]. Risk factors for vertebral osteomyelitis include increased age, state of immunosuppression, diabetes mellitus, long-term corticosteroid usage, malignancy, malnutrition, and IV drug usage [1,2].

Vertebral osteomyelitis usually involves the vertebral body, with only 5% of cases involving the posterior structures of the spine; this is largely because of the superior blood supply of the vertebral bodies. Infection can spread to paraspinal tissues, nerve roots, the epidural space, and the intradural space, creating inflammation, abscesses, and destruction of soft tissue and bone [1]. Intervertebral disc space infections (spinal discitis) likely begin in contiguous end plates, with the infection spreading to the disc secondarily. However, the origin of the infection in children is debated, with some experts believing that spinal discitis in children is caused by partial dislocation of the epiphysis [2].

Vertebral osteomyelitis presents largely with nonspecific symptoms and is therefore difficult to diagnose at early stages. Back pain is the most common presenting symptom, followed by fever (present in 35% to 60% of cases) [1]. Though pain may initially be absent or diffuse, it tends to localize as the infection progresses, with lumbar vertebrae being the most common site of infection, followed by thoracic and cervical vertebrae, respectively. Chills, weight loss, muscle spasms, painful or difficult urination, neurologic impairment, and tenderness to palpation can also be present [1,2]. At late stages, vertebral osteomyelitis can lead to significant morbidity, including spinal deformity, paraplegia, and death [1].

Diagnosis of vertebral infection can be made with a combination of blood tests, imaging, and biopsy. The most sensitive blood tests are the erythrocyte sedimentation rate (ESR) and C-reactive protein (CRP) serum tests, with sensitivity ranging from 94% to 100%. These inflammatory markers are also used to track the progress of treatment. Blood cultures are also indicated for febrile back pain, and stable patients should not receive antibiotics until blood culture results are complete. Computed tomography (CT)-guided biopsy sampling of the vertebra or disc space can be used to identify the pathogen in cases where imaging supports infection, but blood cultures return negative [1]. Imaging is also indicated to identify the location and appearance of the infection. MRI with and without gadolinium contrast enhancement is the gold standard in identifying spinal infection, with a sensitivity of 91% and a specificity of 77% [2,3]. CT scans can also be utilized to determine the amount of bone destruction [2].

The diagnosis and treatment of vertebral osteomyelitis are complex and best managed by an interprofessional team. The initial treatment in all patients is antibiotics for a minimum of six weeks. Surgery is recommended only in complex cases, in the presence of neurological deficits, or with the failure of antibiotics to eliminate the infection. After treatment, serial imaging is necessary to ensure healing and physical rehabilitation is recommended to restore muscle strength [1].

## Case presentation

**Figure 1:**
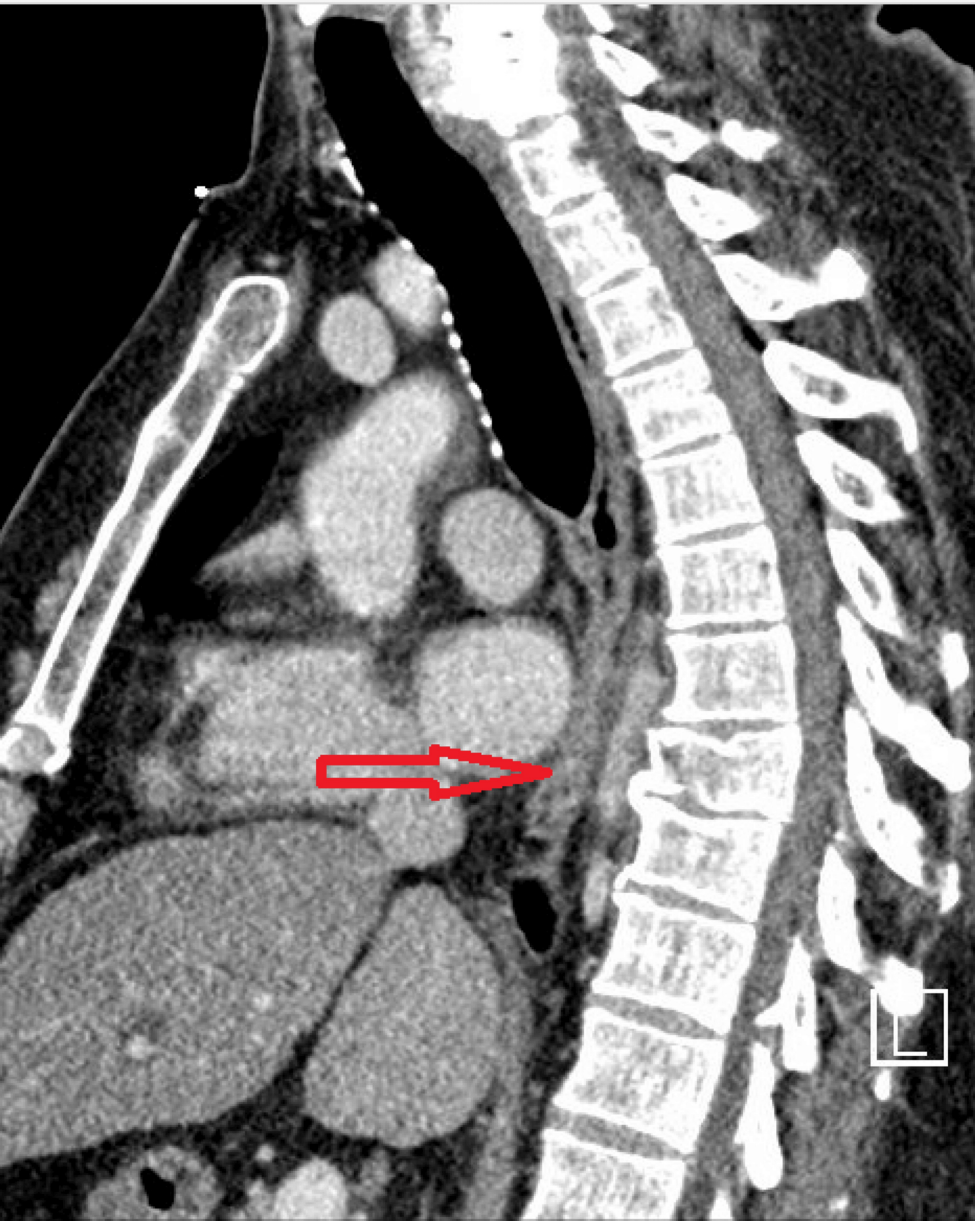
CT imaging of T8 fracture on the day of fracture.

The patient was a 54-year-old male with a history of poorly controlled type 2 diabetes mellitus (A1c 12.1), primary hypertension, splenectomy, and tobacco abuse who was initially involved in a motorcycle accident that resulted in a T8 spinal fracture (Figure 1), as well as several other fractures in the pelvis and lower extremities. He received multiple surgeries for many of the fractures but was treated conservatively for the T8 fracture. The patient’s hospital course was complicated by methicillin-resistant *S. aureus* (MRSA) bacteremia and the patient was discharged to inpatient rehabilitation to complete a four-week course of daptomycin.

**Figure 2:**
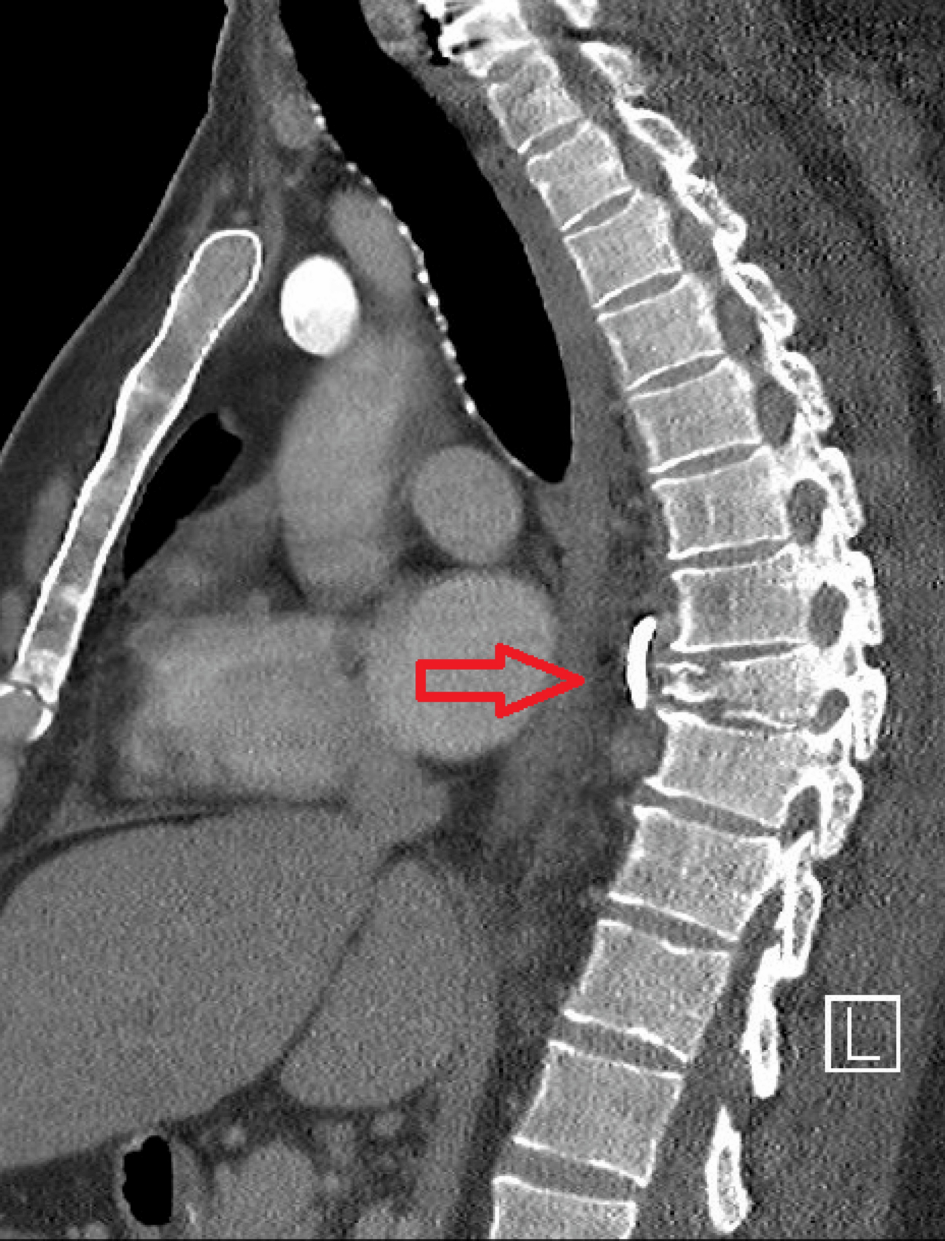
CT imaging showing paravertebral fluid collection around the T8 fracture.

About a week after the completion of the antibiotic course, the patient presented to the emergency department with worsening fever, chills, body aches, and intractable mid-back pain. A CT of the abdomen and pelvis with IV contrast showed a paravertebral fluid collection around the fracture at T8(Figure 2). Labs once again showed bacteremia with MRSA. The interventional radiology team placed a drain a couple of days later, though his blood cultures continued to test positive for a couple more weeks, at which point a PET CT revealed hypermetabolic activity around T8-T9.

**Figure 3:**
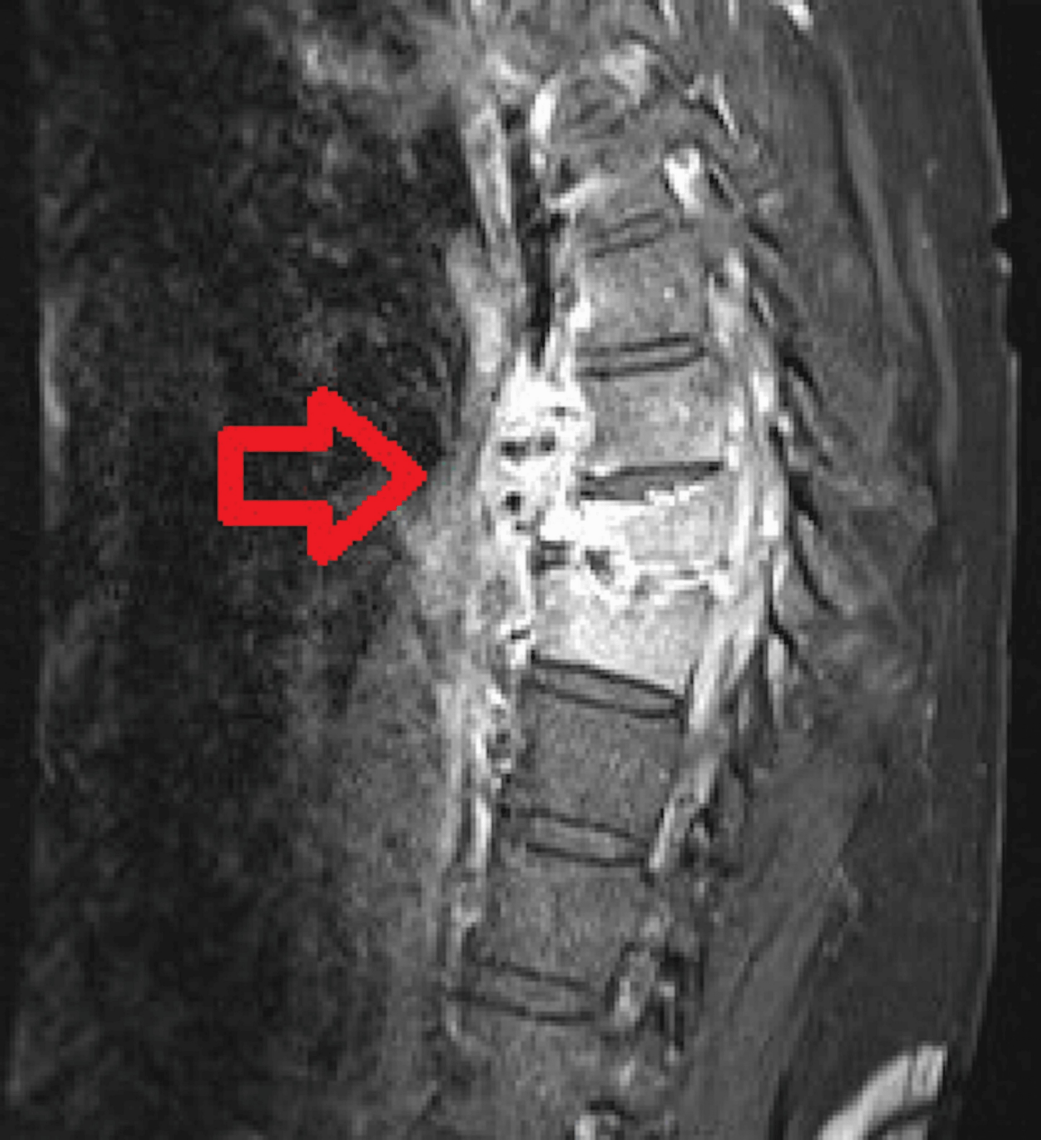
MRI acquired the day before surgery.

The Infectious Disease team was concerned that the patient was not clearing the bacteremia for three weeks despite appropriate and maximal antibiotics, which indicated that the source of infection was not controlled. As a result, they recommended surgery involving the removal of the infected bone and the epidural abscess (debridement) to achieve source control. However, the Neurosurgery team did not believe the patient would be able to tolerate such a large surgery given his condition, age, and comorbidities. They also believed that the patient was not clearing the infection due to spinal instability, rather than source control. The Neurosurgery team performed a minimally invasive open reduction internal fixation of the T8 fracture with segmental percutaneous pedicle and rod fixation spanning T5-T11 bilaterally. MRI of the vertebral column from the day before surgery is shown in Figure 3. Cobalt-chromium rods and titanium screws were implanted, with screws being placed above and below areas of infected bone to reduce the risk of infection persistence.

**Figure 4:**
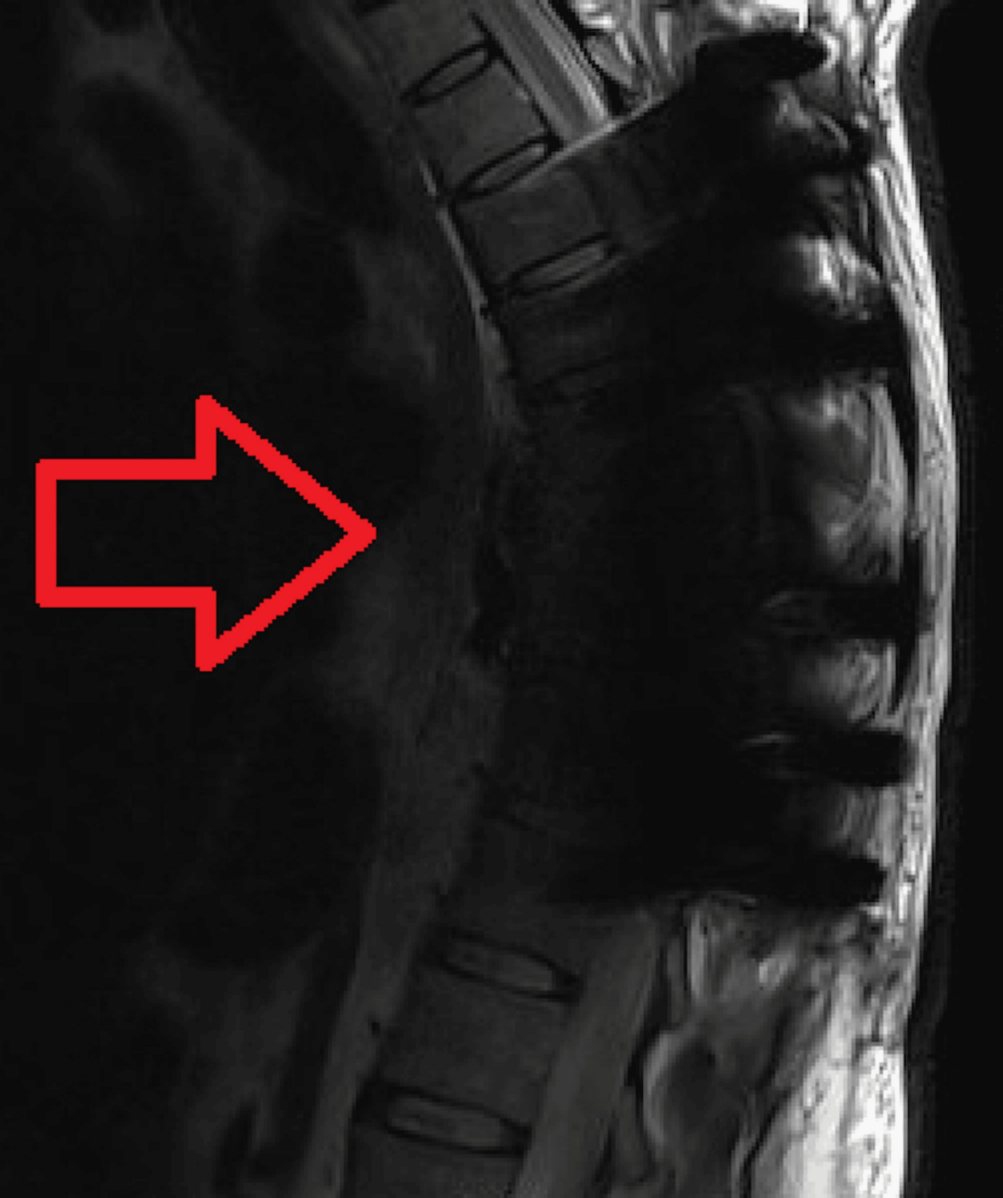
MRI acquired before discontinuation of IV antibiotics.

Within a week after surgery, the patient’s numerous blood cultures all returned negative. The patient’s daptomycin was switched to vancomycin due to eosinophilia, and he was continued on IV vancomycin, 600 mg Q8hr, and oral rifampin for six weeks. Eight days before the end of the antibiotic treatment, an MRI was completed which, though limited by a metallic susceptibility artifact from the hardware, showed no signs of residual infection (Figure 4). The case was discussed with the Infectious Disease team, who recommended discontinuing the IV antibiotic regimen. The patient was discharged with doxycycline 100 mg BID and has followed up with the Infectious Disease team in the clinic. They have since recommended continuous doxycycline administration, and the patient has been following up without complications for about a year.

## Discussion

IV antibiotic or antifungal therapy is the mainstay of spinal infection treatment, though regimens have been proposed that utilize oral administration [1,2]. Patients generally undergo medical therapy for a minimum of six to eight weeks [2]. Most authorities recommend six weeks of therapy, with research showing longer courses of treatment are not more effective in typical cases [1]. Immobilization is common and recommended in cases of significant pain or potential for instability [2].

Surgical treatment for osteomyelitis may be indicated in cases of significant bone destruction causing spinal instability, neurological deficits, large epidural abscess formation, intractable back pain, sepsis with clinical toxicity caused by an abscess that is unresponsive to antibiotics, failure of biopsy to obtain needed cultures, or failure of antibiotics alone to eradicate the infection [1,2]. Goals of surgery can include debridement of the infected tissue, increasing blood flow to promote healing, restoration of spinal stability with the use of instrumentation to fuse the unstable spine, and restoration of neurological function [2]. In this case, spinal instability and failure of antibiotics to eliminate the infection led to surgery, with the goal of restoring spinal stability. Like other forms of osteomyelitis, debridement plays an important role in surgical management [4]. However, in this case, the patient was unlikely to tolerate a debridement procedure, so the surgical focus was to stabilize the infected vertebrae to promote clearing of the infection.

Cobalt-chromium rods and titanium screws were implemented in this case. Research comparing the antibacterial properties of titanium with cobalt-chromium is somewhat conflicting. One study found that the cobalt-chromium alloy has a superior antibacterial effect than titanium alloys [5]. However, another study found that titanium spinal implants had significantly lower rates of *S. aureus* biofilm formation than cobalt-chromium implants [6].

Similarly, research comparing the antibacterial properties of titanium with stainless steel is also conflicting. An older experimental study showed that titanium implants had a lower risk of infection compared to those of stainless steel [7]. Another study shows that titanium is more resistant to infection and biofilm formation than stainless steel and hydroxyapatite-coated steel in Kirschner wires [8]. However, other research shows that titanium can be associated with a significant rise in implant-associated infections (IAIs) and that it does not have inherent antibacterial properties [9,10]. A systematic review showed that no proven advantage can be seen in titanium over stainless steel with regard to IAIs in contemporary literature [11]. Another large review also showed that, despite slight tendencies, there is no proof of titanium alloy being favorable in the susceptibility of IAI [12]. Considerable debate remains surrounding the influence of implant material on IAI, and future research investigating the susceptibility of these metals to infection is necessary to fully answer this question [11,12]. It is important to keep in mind that implants that cause less soft tissue damage and preserve perfusion of the periosteum show significant benefits regarding IAI [11,12].

To reduce the risk of infections, a variety of antibacterial agents can be incorporated on the surface of the titanium implant, and other surface modifications can be made as well [9,13]. Research is also examining the use of UV irradiation to reduce infection rates [13]. Introducing metallic implants in the setting of infection can be controversial, but surgeons are increasingly acknowledging that instrumentation can help the body fight infection rather than interfere with the healing process [4].

Early researchers believed that surgical treatment of osteomyelitis increased the likelihood of infection persistence or recurrence [1]. Internal fixation in the setting of acute infection started gaining acceptance in the 1990s, with an increasing number of surgeons reporting their series of surgical treatments with excellent results [4]. The utilization of metallic implants in the setting of infection is controversial, and the notion that implants can act as a foreign body through which bacteria can attach, and form biofilms is rational [4].

Some data have shown a 2%-9% increased risk of infection after spinal instrumentation [4]. One study found that, though surgery for vertebral osteomyelitis significantly improved neurologic function and decreased pain, it does have a high incidence of adverse events [14]. However, a more extensive review argued that the use of metallic implants in an infected area of the spine is safe and does not lead to the persistence or recurrence of infection [4]. Recent literature suggests that surgery does not increase adverse clinical outcomes in cases of vertebral osteomyelitis [1].

Though our understanding of the crucial role of mechanical stability with regard to bone healing has greatly advanced, much has remained unknown about the role of mechanical stability in the context of fracture-related infection (FRI). A few preclinical animal models remain the basis for current surgical decision-making [15]. However, there is consensus that stability is important in treating FRI because instability leads to a harmful cycle of positive feedback in which fracture instability impairs neovascularity, worsening soft-tissue trauma and osteolysis, which in turn promotes bacterial proliferation and compromises host responses. In turn, this leads to further osteolysis and instability [15]. 

This case report emphasizes how minimally invasive surgery can be successfully used for stabilization when antibiotics alone fail to treat osteomyelitis. Limitations include a relatively short length of follow-up (approx. one year), limited generalizability to the population, and lack of comparative data analysis. Future studies examining the effectiveness of spinal fixation and the antibacterial properties of implant materials could provide valuable insights into the remaining questions raised in this report.

## Conclusions

This case report highlights the importance of spinal stability in treating vertebral osteomyelitis. Though some data have shown that spinal instrumentation can lead to infection persistence, recent literature suggests that surgery and metallic implants do not increase adverse clinical outcomes in cases of vertebral osteomyelitis. Understanding whether titanium reduces the risk of bacterial infection over cobalt-chromium or stainless steel requires further research. Instability disrupts the healing process by causing a cycle of impaired neovascularity, soft-tissue trauma, and osteolysis.
